# Supplementary material for: Sedimentation and drag in drifting macrophytes and plastic objects: a model
Source: Sci Rep. 2025 Nov 27;15:43088. doi: 10.1038/s41598-025-28893-8 (PMC12675491; doi:10.1038/s41598-025-28893-8)
Supplement: Supplementary file 1 — Supplementary Material 1 [file 41598_2025_28893_MOESM1_ESM.pdf]

Online supplement to:

Sedimentation and drag in drifting macrophytes and plastic objects: A model

Authors: Friederike Gronwald[1#], Florian Weinberger [1#*], Tjeerd J. Bouma[2], Rolf Karez[3]

[#] **These authors contributed equally to this work**

[*]**Corresponding author:** Florian Weinberger, GEOMAR Helmholtz Centre for Ocean Research Kiel, Wischhofstraße 1-3, 24148 Kiel, Germany, telephone: +49 431 600 4516, E-Mail: fweinberger@geomar.de

Affiliations

[1] Department of Marine Ecology, GEOMAR Helmholtz Centre for Ocean Research Kiel, 24148 Kiel, Germany.

[2] NIOZ Royal Netherlands Institute for Sea Research, Department of Estuarine and Delta Systems, 4401 NT Yerseke, the Netherlands.

[3] State Agency for the Environment Schleswig-Holstein, 24220 Flintbek, Germany.

Table S1: Shape descriptors of the investigated specimens. Non-buoyant specimens were grouped and used either for development or for testing of the sedimentation models, while buoyant specimens could obviously not be used for this purpose. Weight is the blotting weight [g], V is the volume [cm³], d_n [cm] is the nominal diameter. c [mm], a and b [cm] are thallus diameters in the shortest, longest and intermediate mutually perpendicular axes, respectively, that were used to calculate the Corey shape factor S_f (see Eq. 2). Bold values listed under c and a b were measured directly, while non-bold values listed under c and a b were determined by division of V by the bold value. Figure refers to pictures in the online supplement. ρ_{H2O} and ν are seawater density and kinematic viscosity during measurements of the mean sedimentation velocity ω.

Specimen	Group	Weight [g]	V [cm³]	d_n [cm]	c [mm]	a b [cm²]	S_f	Figure	ρ_{sw} [kg/m³]	ν [m² s⁻¹]	ω [m s⁻¹]
Acrosiphonia centralis 36	Modeling	12.98	12.25	2.86	**0.102**	1205.7	0.00029	S4	1009.446	1.0494E-06	0.0208
Ahnfeltia plicata 47-1	Modeling	0.66	0.50	0.98	**0.455**	11.0	0.01371	S5	1009.862	1.0917E-06	0.0347
Ahnfeltia plicata 47-2	Modeling	7.07	5.63	2.21	**0.492**	114.4	0.00460	S5	1009.862	1.0917E-06	0.0466
Bryopsis hypnoides 1-2	Modeling	0.95	0.88	1.19	**0.104**	84.5	0.00113	S4	1009.862	1.0917E-06	0.0144
Coccotylus truncatus 24-2-1	Modeling	0.50	0.45	0.95	0.142	**31.6**	0.00253	S5	1009.710	1.0915E-06	0.0277
Delesseria sanguinea 26-2	Modeling	1.62	1.25	1.34	0.144	**86.6**	0.00155	S5	1009.446	1.0494E-06	0.0181
Fucus serratus 9	Modeling	6.15	5.50	2.19	0.492	**111.8**	0.00465	S4	1009.446	1.0494E-06	0.0290
Fucus vesiculosus 54	Modeling	17.88	17.00	3.19	0.653	**260.2**	0.00405	S4	1009.446	1.0494E-06	0.0256
Furcellaria lumbricalis 43-1	Modeling	1.31	1.08	1.27	**0.606**	17.9	0.01433	S5	1009.564	1.0727E-06	0.0450
Furcellaria lumbricalis 44-1	Modeling	2.39	2.00	1.56	**0.530**	37.8	0.00862	S5	1009.564	1.0727E-06	0.0525
Gracilaria vermiculophylla 16-1	Modeling	0.45	0.42	0.93	**0.351**	12.1	0.01007	S5	1009.710	1.0915E-06	0.0306
Gracilaria vermiculophylla 49	Modeling	5.23	5.00	2.12	**0.279**	179.2	0.00208	S5	1009.488	1.0726E-06	0.0237
Kornmannia leptoderma 1-1	Modeling	0.29	0.27	0.81	0.052	**52.4**	0.00073	S4	1009.724	1.0835E-06	0.0084
Polysiphonia stricta 37-2-1	Modeling	1.69	1.58	1.45	**0.094**	168.8	0.00072	S4	1009.745	1.0862E-06	0.0124
Saccharina latissima rhizoid K9B	Modeling	0.98	0.70	1.10	**0.815**	8.6	0.02779	S4	1013.900	1.1939E-06	0.0492
Spermothamnion repens 14	Modeling	0.47	0.40	0.91	**0.078**	51.4	0.00109	S5	1009.862	1.0917E-06	0.0169
Ulva clathrata 21-1	Modeling	0.30	0.25	0.78	**0.323**	7.7	0.01163	S4	1009.710	1.0915E-06	0.0097

Table S1, continued.

Specimen	Group	Weight [g]	V [cm³]	d_n [cm]	c [mm]	a b [cm²]	S_f	Figure	ρ_{sw} [kg/m³]	ν [m² s⁻¹]	ω [m s⁻¹]
Ulva gigantea 10	Modeling	5.98	5.50	2.19	0.079	**698.2**	0.00030	S4	1009.446	1.0494E-06	0.0149
Ulva linza 8-1	Modeling	0.23	0.20	0.73	0.151	**13.3**	0.00413	S4	1009.745	1.0862E-06	0.0119
Zostera marina leaf 40-2	Modeling	0.16	0.13	0.62	0.168	**7.4**	0.00618	S4	1009.446	1.0494E-06	0.0108
Ahnfeltia plicata K1	Testing	1.26	1.00	1.24	**0.190**	52.8	0.00261	S8	1013.900	1.1939E-06	0.0287
Ceramium virgatum 6-2	Testing	2.41	2.38	1.66	**0.079**	299.5	0.00046	S7	1009.745	1.0862E-06	0.0119
Ceramium virgatum 7-2	Testing	1.47	1.25	1.34	**0.186**	67.3	0.00226	S7	1009.710	1.0915E-06	0.0132
Ceramium virgatum 8-2	Testing	0.91	0.88	1.19	**0.146**	60.6	0.00187	S7	1009.745	1.0862E-06	0.0122
Ceramium virgatum K6	Testing	0.12	0.10	0.58	**0.054**	18.4	0.00127	S7	1013.900	1.1939E-06	0.0100
Cladophora flexuosa 34	Testing	4.97	4.50	2.05	**0.152**	296.4	0.00088	S6	1013.900	1.1939E-06	0.0087
Cladophora sp. K3	Testing	0.11	0.10	0.58	**0.076**	13.1	0.00211	S6	1013.900	1.1939E-06	0.0149
Coccotylus truncatus 24-2-2	Testing	0.39	0.35	0.88	0.354	**10.0**	0.01121	S8	1009.598	1.0967E-06	0.0276
Delesseria sanguinea 27	Testing	18.74	16.50	3.16	0.376	**438.3**	0.00180	S7	1009.446	1.0494E-06	0.0281
Delesseria sanguinea 27-2-2	Testing	10.69	9.41	2.62	0.297	**317.2**	0.00167	S7	1009.598	1.0967E-06	0.0282
Fucus serratus 12	Testing	9.90	8.88	2.57	0.512	**173.2**	0.00389	S6	1009.446	1.0494E-06	0.0384
Fucus serratus K5	Testing	9.37	8.30	2.51	0.443	**187.5**	0.00323	S6	1013.900	1.1939E-06	0.0321
Furcellaria lumbricalis 41-1	Testing	2.00	1.58	1.45	**0.659**	24.0	0.01345	S8	1009.564	1.0727E-06	0.0499
Furcellaria lumbricalis K8	Testing	0.73	0.60	1.05	**0.422**	14.2	0.01118	S8	1013.900	1.1939E-06	0.0380
Gracilaria vermiculophylla 26-1	Testing	4.64	4.25	2.01	**0.253**	168.1	0.00195	S8	1009.446	1.0494E-06	0.0226
Gracilaria vermiculophylla 49-2	Testing	2.62	2.50	1.68	**0.328**	76.4	0.00375	S8	1009.598	1.0967E-06	0.0209
Kornmannia leptoderma 4-2	Testing	0.11	0.10	0.58	0.038	**26.2**	0.00074	S6	1009.724	1.0835E-06	0.0080
Polysiphonia stricta 37-2-2	Testing	1.06	1.00	1.24	**0.110**	90.9	0.00115	S7	1009.598	1.0967E-06	0.0118
Polysiphonia stricta 39-2	Testing	1.28	1.25	1.34	**0.117**	106.5	0.00114	S7	1009.745	1.0862E-06	0.0143
Pyropia leucosticta 32	Testing	0.92	0.88	1.19	0.087	**100.7**	0.00087	S7	1009.488	1.0726E-06	0.0111
Rhodomela confervoides 4-1	Testing	3.60	3.50	1.88	**0.385**	90.9	0.00404	S7	1009.724	1.0835E-06	0.0200
Rhodomela confervoides 4-1-2	Testing	2.47	2.40	1.66	**0.373**	64.3	0.00465	S7	1009.598	1.0967E-06	0.0201
Saccharina latissima phylloid K9A	Testing	2.01	1.60	1.45	0.263	**60.9**	0.00337	S6	1013.900	1.1939E-06	0.0167

Table S1, continued.

Specimen	Group	Weight [g]	V [cm³]	d_n [cm]	c [mm]	a b [cm²]	S_f	Figure	ρ_{sw} [kg/m³]	v [m² s⁻¹]	ω [m s⁻¹]
Spermothamnion repens 16-2	Testing	0.24	0.20	0.73	**0.050**	39.7	0.00080	S7	1009.710	1.0915E-06	0.0164
Ulva clathrata 24-1	Testing	0.19	0.18	0.69	**0.245**	7.1	0.00918	S6	1009.710	1.0915E-06	0.0074
Ulva linza 6-1	Testing	0.52	0.43	0.93	0.156	**27.2**	0.00300	S6	1009.745	1.0862E-06	0.0330
Vertebrata fucoides K2	Testing	0.29	0.20	0.73	**0.077**	25.8	0.00152	S6	1013.900	1.1939E-06	0.0139
Zostera marina leaf 40-1	Testing	0.48	0.43	0.93	0.222	**19.1**	0.00509	S6	1009.446	1.0494E-06	0.0137
Zostera marina leaf K7	Testing	0.23	0.20	0.73	0.156	**12.8**	0.00435	S6	1013.900	1.1939E-06	0.0168
Ascophyllum nodosum 21	Buoyant	8.39	8.63	2.54	1.756	**49.1**	0.02505	S10	1009.446	1.0494E-06	0
Ascophyllum nodosum 29	Buoyant	46.10	59.50	4.84	3.384	**175.8**	0.02552	S10	1009.446	1.0494E-06	0
Ascophyllum nodosum 2	Buoyant	3.38	4.33	2.02	2.138	**20.3**	0.04751	S10	1009.446	1.0494E-06	0
Ascophyllum nodosum 46	Buoyant	1.85	2.58	1.70	2.307	**11.2**	0.06893	S10	1009.446	1.0494E-06	0
Ascophyllum nodosum 5	Buoyant	4.81	4.83	2.10	1.747	**27.7**	0.03323	S10	1009.446	1.0494E-06	0
Ascophyllum nodosum 68	Buoyant	12.17	13.00	2.92	1.820	**71.4**	0.02153	S10	1009.446	1.0494E-06	0
Chorda filum 41-2	Buoyant	0.61	0.88	1.19	1.230	**7.1**	0.04614	S9	1009.564	1.0727E-06	0
Chorda filum 42-2	Buoyant	0.98	1.17	1.31	1.086	**10.7**	0.03313	S9	1009.745	1.0862E-06	0
Chorda filum 43-2	Buoyant	0.42	0.58	1.04	1.025	**5.7**	0.04296	S9	1009.564	1.0727E-06	0
Chorda filum 44-2	Buoyant	0.77	1.42	1.39	1.633	**8.7**	0.05544	S9	1009.564	1.0727E-06	0
Fucus vesiculosus 13	Buoyant	6.70	9.50	2.63	1.192	**79.7**	0.01335	S10	1009.446	1.0494E-06	0
Fucus vesiculosus 15	Buoyant	6.04	6.50	2.32	0.861	**75.5**	0.00990	S10	1009.446	1.0494E-06	0
Fucus vesiculosus 3	Buoyant	7.80	8.88	2.57	1.185	**74.9**	0.01370	S10	1009.488	1.0726E-06	0
Fucus vesiculosus 35	Buoyant	6.17	7.38	2.42	1.102	**66.9**	0.01347	S10	1009.488	1.0726E-06	0
Fucus vesiculosus 38	Buoyant	4.82	5.50	2.19	1.018	**54.0**	0.01385	S10	1009.446	1.0494E-06	0
Fucus vesiculosus 51	Buoyant	10.33	10.75	2.74	1.237	**86.9**	0.01327	S10	1009.446	1.0494E-06	0
Fucus vesiculosus 53	Buoyant	14.70	17.50	3.22	0.905	**193.3**	0.00651	S10	1009.446	1.0494E-06	0
Ulva compressa 48-2	Buoyant	0.89	1.00	1.24	0.142	**70.3**	0.00170	S10	1009.862	1.0917E-06	0
Zostera marina 25	Buoyant	3.69	3.75	1.93	**0.171**	219.0	0.00116	S9	1009.446	1.0494E-06	0
Zostera marina 37-1	Buoyant	3.15	3.50	1.88	**0.298**	117.5	0.00275	S9	1009.745	1.0862E-06	0
Zostera marina 39-1	Buoyant	0.37	0.50	0.98	0.311	**16.1**	0.00774	S9	1009.745	1.0862E-06	0

Table S1, continued.

Specimen	Group	Weight [g]	V [cm³]	d_n [cm]	c [mm]	a b [cm²]	S_f	Figure	ρ_{sw} [kg/m³]	v [m² s⁻¹]	ω [m s⁻¹]
Zostera marina 45	Buoyant	2.84	3.50	1.88	**0.498**	70.3	0.00594	S9			0
Zostera marina 48-1	Buoyant	1.69	2.00	1.56	**0.300**	66.8	0.00367	S9	1009.862	1.0917E-06	0
Zostera marina 69	Buoyant	1.71	1.88	1.53	0.856	**21.9**	0.01830	S9			0
Disc 40-1	Plastic	0.25	0.18	0.71	0.14	**12.7**	0.00406	S11	998.15	9.7629E-07	0.0225
Disc 59-1	Plastic	0.55	0.40	0.92	0.15	**27.6**	0.00279	S11	998.15	9.7629E-07	0.0187
Disc 59-2	Plastic	0.25	0.18	0.71	0.15	**12.5**	0.00417	S11	998.15	9.7629E-07	0.0260
Disc 70-1	Plastic	0.8	0.59	1.04	0.15	**39.9**	0.00236	S11	998.15	9.7629E-07	0.0217
Disc 70-2	Plastic	0.56	0.41	0.92	0.15	**27.7**	0.00280	S11	998.15	9.7629E-07	0.0234
Disc 70-3	Plastic	0.25	0.18	0.71	0.15	**12.5**	0.00417	S11	998.15	9.7629E-07	0.0507
Disc 84-1	Plastic	1.13	0.83	1.17	0.15	**55.4**	0.00202	S11	998.15	9.7629E-07	0.0283
Disc 84-4	Plastic	0.26	0.19	0.71	0.15	**12.7**	0.00413	S11	998.15	9.7629E-07	0.0298
Ball 1	Plastic	33.62	33.51	4	**26.67**	12.6	1	S11	998.37	9.92893E-07	0.0971
Ball 2	Plastic	33.97	33.51	4	**26.67**	12.6	1	S11	998.37	9.92893E-07	0.1622
Ball 3	Plastic	35.17	33.51	4	**26.67**	12.6	1	S11	998.37	9.92893E-07	0.2536
Net-large	Plastic	1.13	1	1.24	**0.13**	80.0	0.00140	S11	998.37	9.92893E-07	0.0493
Net-small	Plastic	1.02	0.8	1.15	**0.13**	64.0	0.00156	S11	998.37	9.92893E-07	0.0605
Rubberband-small	Plastic	0.38	0.27	0.8	**1.34**	2.0	0.09510	S11	998.37	9.92893E-07	0.0769
Rubberband-medium	Plastic	0.4	0.31	0.84	**1.22**	2.5	0.07627	S11	998.37	9.92893E-07	0.0778
Rubberband-large	Plastic	0.69	0.53	1.0	**1.38**	3.8	0.07045	S11	998.37	9.92893E-07	0.0819

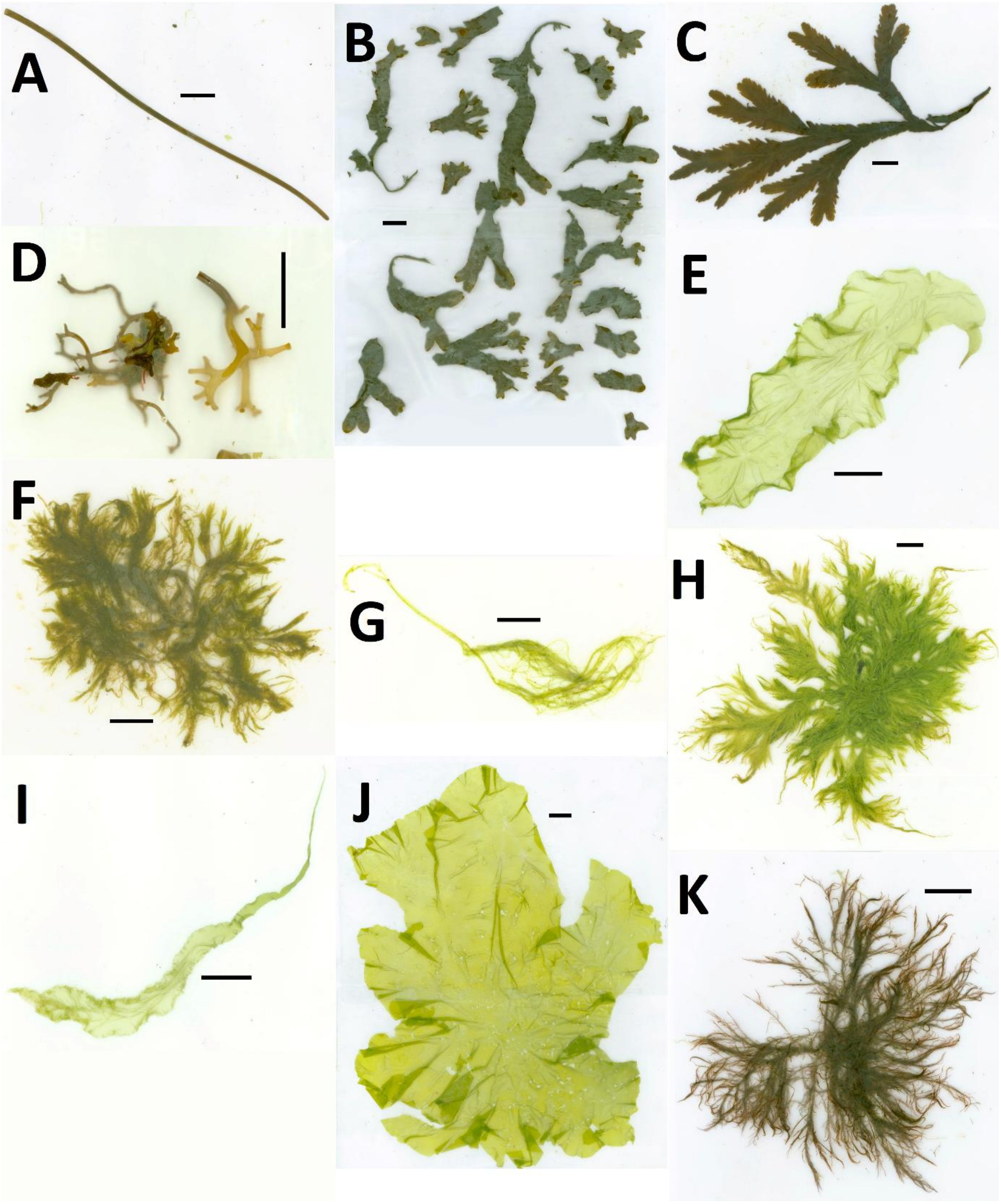

Fig. S1: Specimens of the modeling sample set. (A) *Z. marina* 40-2, (B) *F. vesiculosus* 54, (C) *F. serratus* 9, (D) *S. latissima* rhizoid K9B, (E) *K. leptoderma* 1-1, (F) *B. hypnoides* 1-2, (G) *U. clathrata* 21-1, (H) *A. centralis* 36, (I) *U. linza* 8-1, (J) *U. gigantea* 10, (K) *P. stricta* 37-2-1. Horizontal or vertical scale bars represent a distance of 2 cm, Specimens in B and D were disassembled to facilitate scanning.

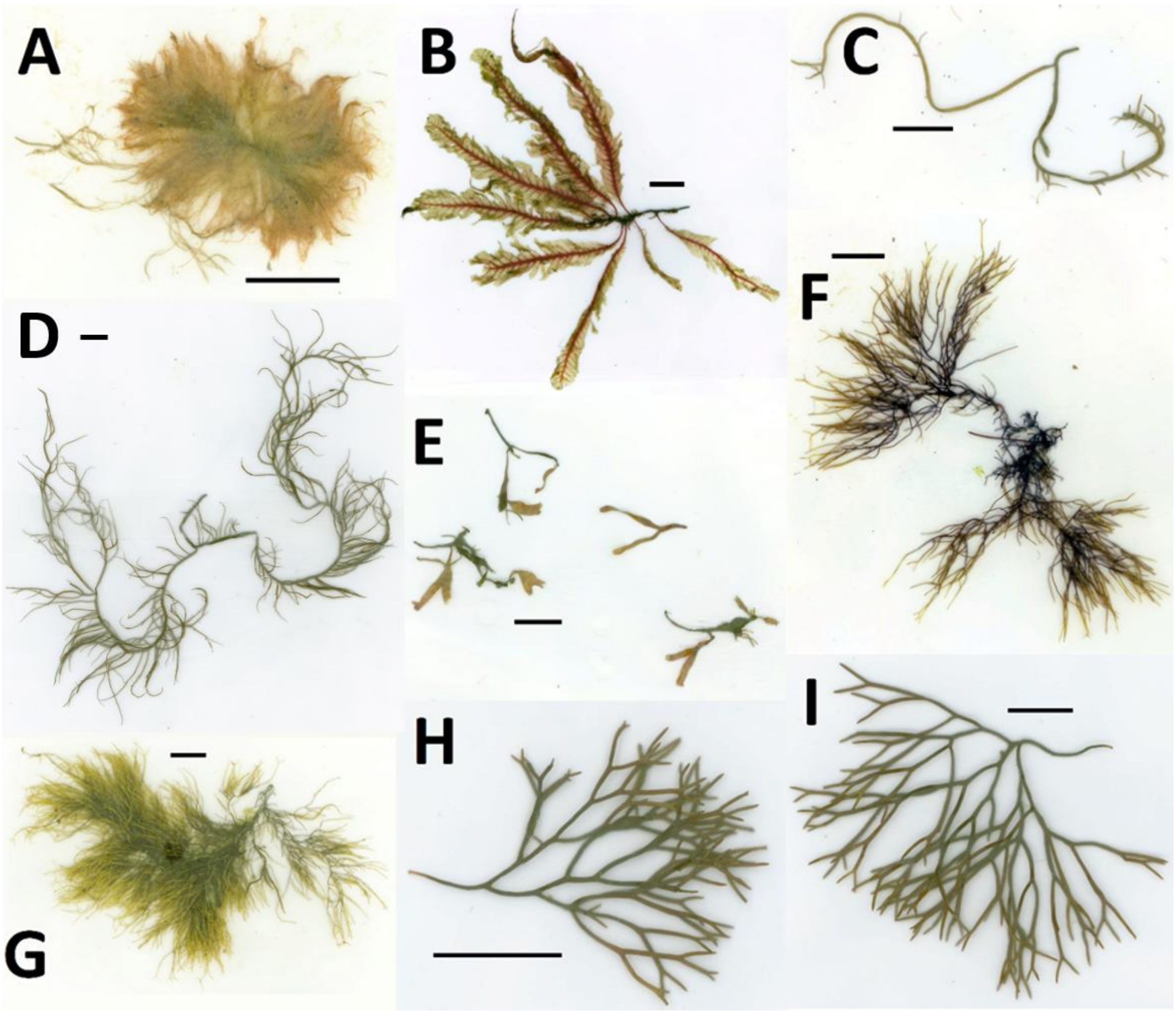

Fig. S2: Specimens of the modeling sample set. (A) *S. repens* 14, (B) *D. sanguinea* 26-2, (C) *G. vermiculophylla* 16-1, (D) *G. vermiculophylla* 49 (E) *C. truncatus* 24-2-1, (F) *A. plicata* 47-1, (G) *A. plicata* 47-2, (H) *F. lumbricalis* 43-1, (I) *F. lumbricalis* 44-1. Horizontal scale bars represent a distance of 2 cm. The specimen in E was disassembled to facilitate scanning.

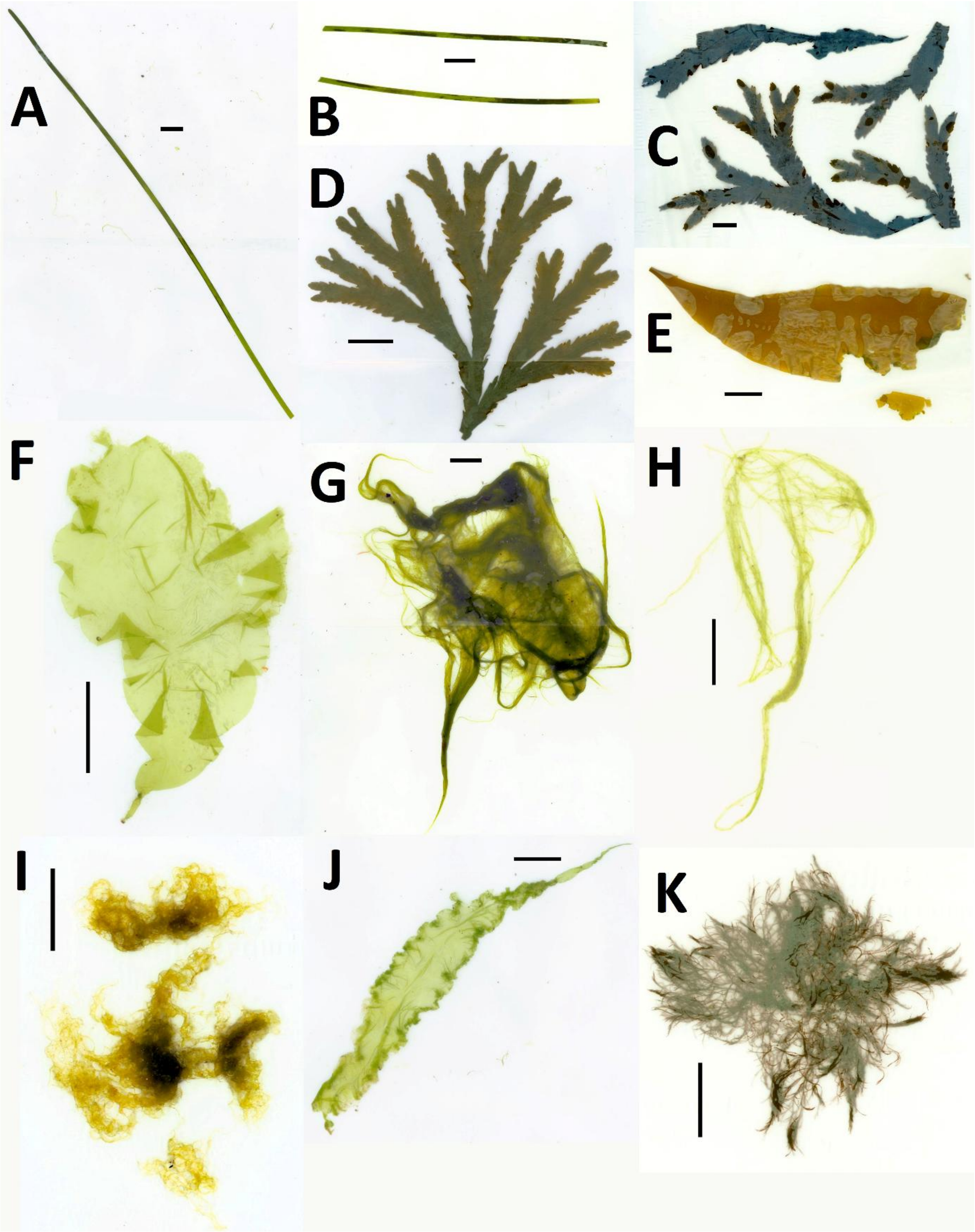

Fig. S3: Specimens of the testing sample set. (A) *Z. marina* 40-1, (B) *Z. marina* K7, (C) *F. serratus* K5, (D) *F. serratus* 12, (E) *S. latissima* phylloid K9A, (F) *K. leptoderma* 4-2, (G) *C. flexuosa* 34, (H) *U. clathrata* 24-1, (I) *Cladophora* sp. K3, (J) *U. linza* 6-1, (K) *V. fucoides* K2. Horizontal or vertical scale bars represent a distance of 2 cm. Specimens in B, C and I were disassembled to facilitate scanning, specimen E broke prior to scanning.

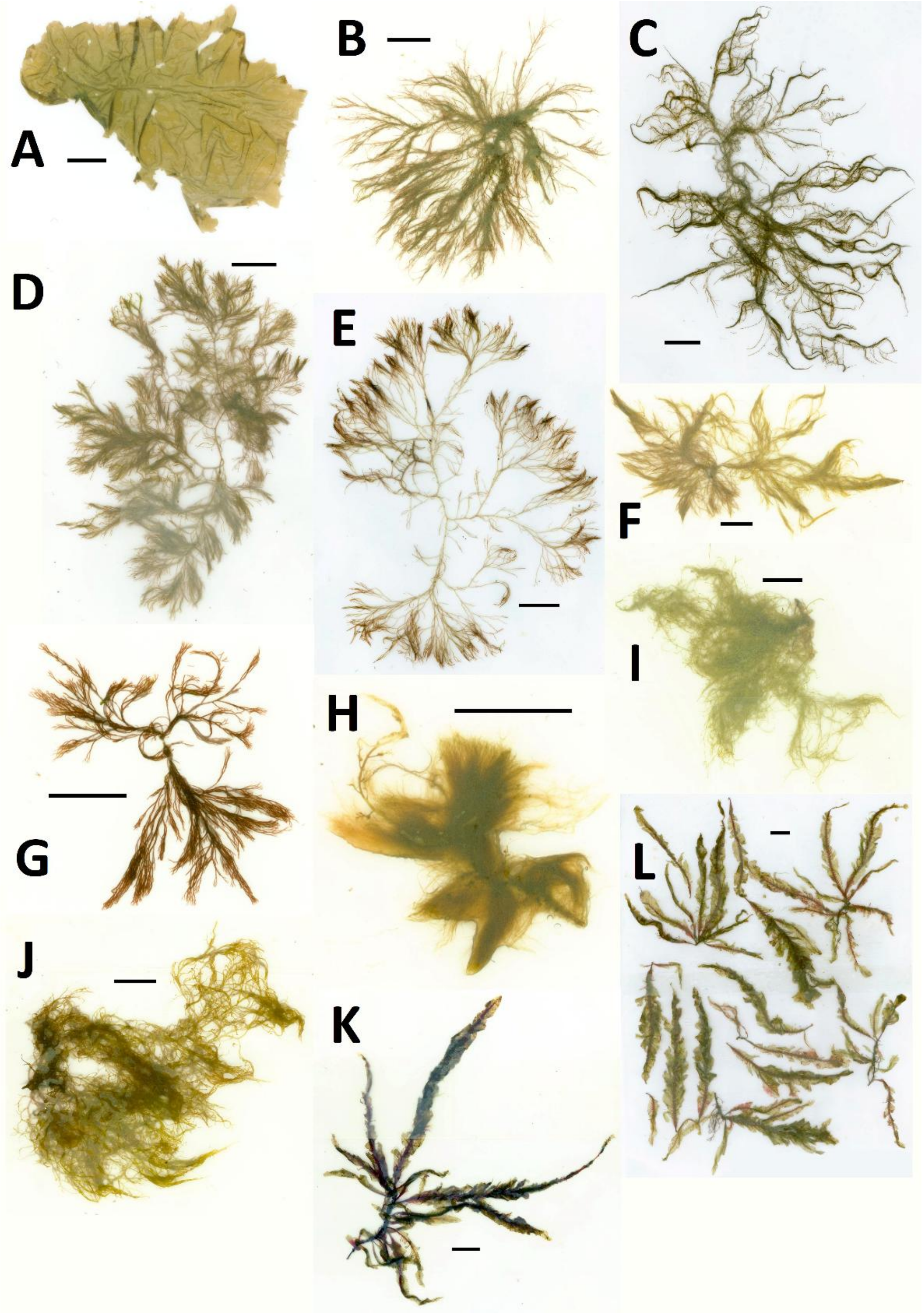

Fig. S4: Specimens of the testing sample set. (A) *P. leucosticta* 32, (B) *P. stricta* 37-2-2, (C) *P. stricta* 39-2, (D) *C. virgatum* 6-2, (E) *C. virgatum* 8-2, (F) *C. virgatum* 7-2, (G) *C. virgatum* K6, (H) *S. repens* 16-2, (I) *R. confervoides* 4-1-2, (J) *R. confervoides* 4-1, (K) *D. sanguinea* 27, (L) *D. sanguinea* 27-2-2. Horizontal or vertical scale bars represent a distance of 2 cm. The specimen in L was disassembled to facilitate scanning.

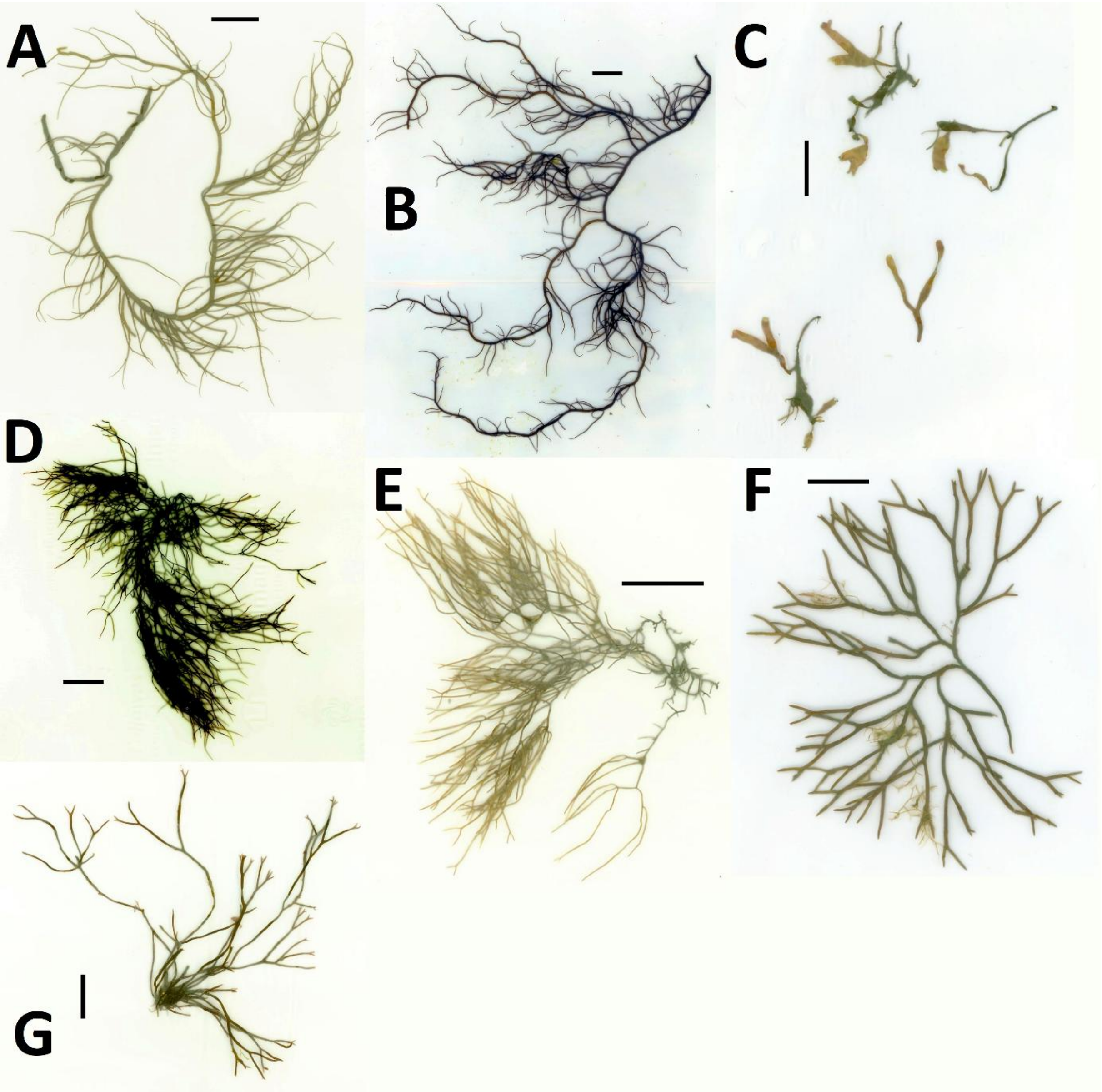

Fig. S5: Specimens of the testing sample set. (A) *G. vermiculophylla* 49-2, (B) *G. vermiculophylla* 26-1, (C) *C. truncatus* 24-2-2, (D) *A. plicata* K1, (E) *A. plicata* 47-1-2, (F) *F. lumbricalis* 41-1, (G) *F. lumbricalis* K8. Horizontal or vertical scale bars represent a distance of 2 cm. The specimen in C was disassembled to facilitate scanning.

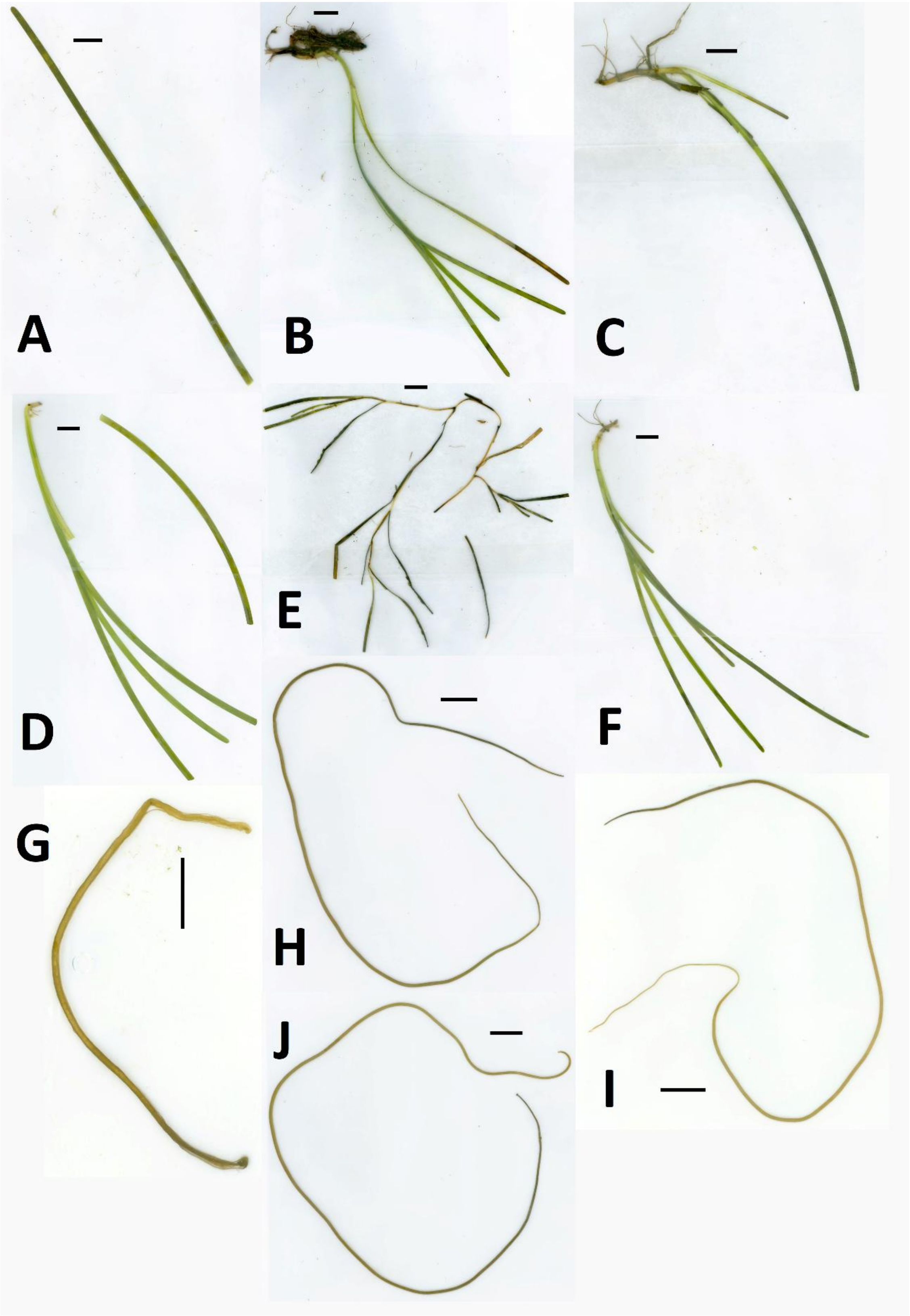

Fig. S6: Positively buoyant specimens. (A) *Z. marina* 39-1, (B) *Z. marina* 37-1, (C) *Z. marina* 69, (D) *Z. marina* K1, (E) *Z. marina* 45, (F) *Z. marina* 48-1, (G) *C. filum* 41-2, (H) *C. filum* 44-2, (I) *C. filum* 43-2, (J) *C. filum* 24-2. Horizontal or vertical scale bars represent a distance of 2 cm. Specimens in D and E broke prior to scanning.

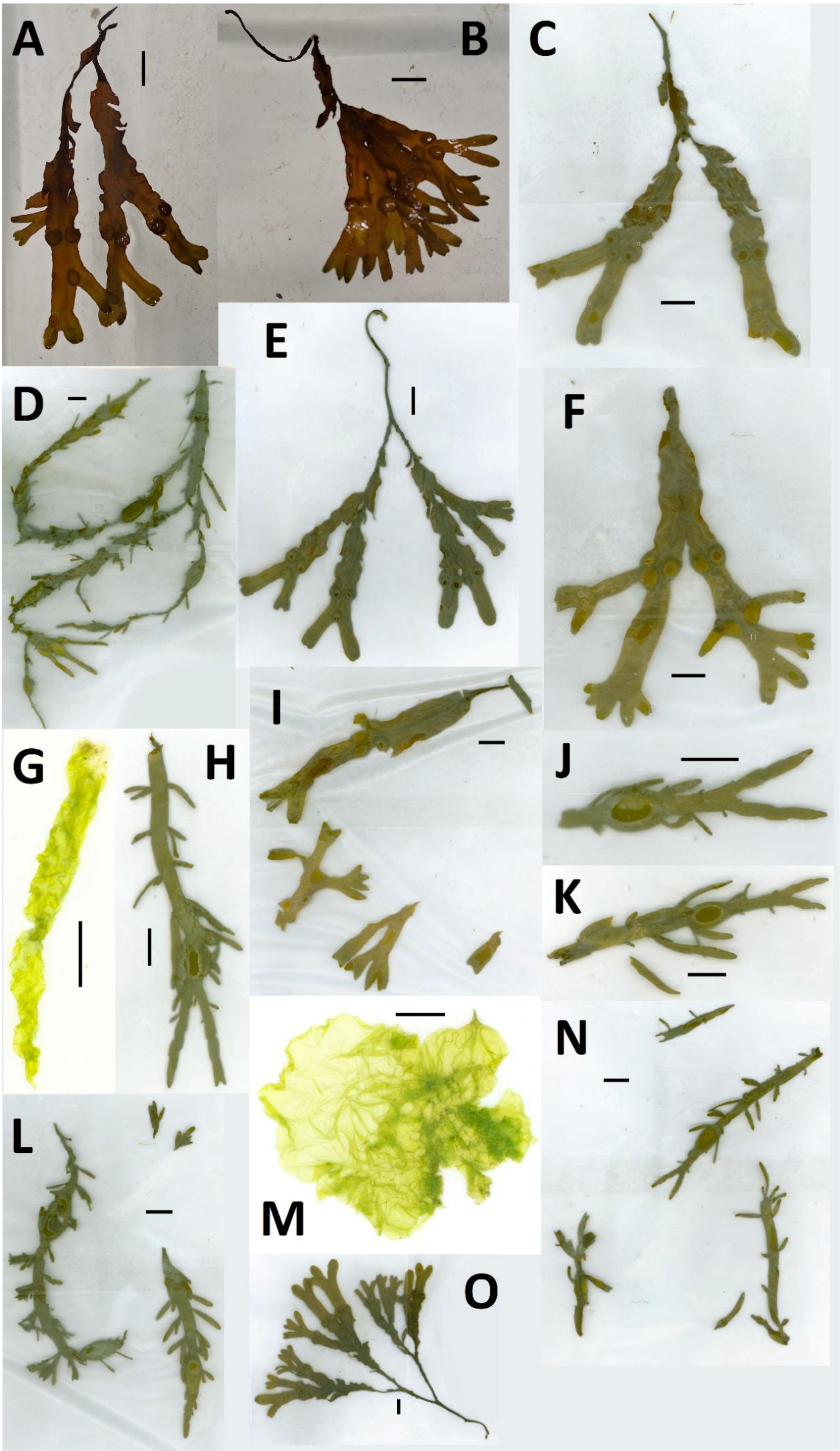
A
B
C
D
E
F
G
H
I
J
K
L
M
N
O

Fig. S7 (previous page): Positively buoyant specimens. (A) *F. vesiculosus* 3, (B) *F. vesiculosus* 35, (C) *F. vesiculosus* 38, (D) *A. nodosum* 29, (E) *F. vesiculosus* 15, (F) *F. vesiculosus* 13, (G) *U. linza* 7-1, (H) *A. nodosum* 5, (I) *F. vesiculosus* 51, (J) *A. nodosum* 46, (K) *A. nodosum* 2, (L) *A. nodosum* 68, (M) *U. compressa* 48-2, (N) *A. nodosum* 21, (O) *F. vesiculosus* 53. Horizontal or vertical scale bars represent a distance of 2 cm. Specimens in I, L and N were disassembled to facilitate scanning.

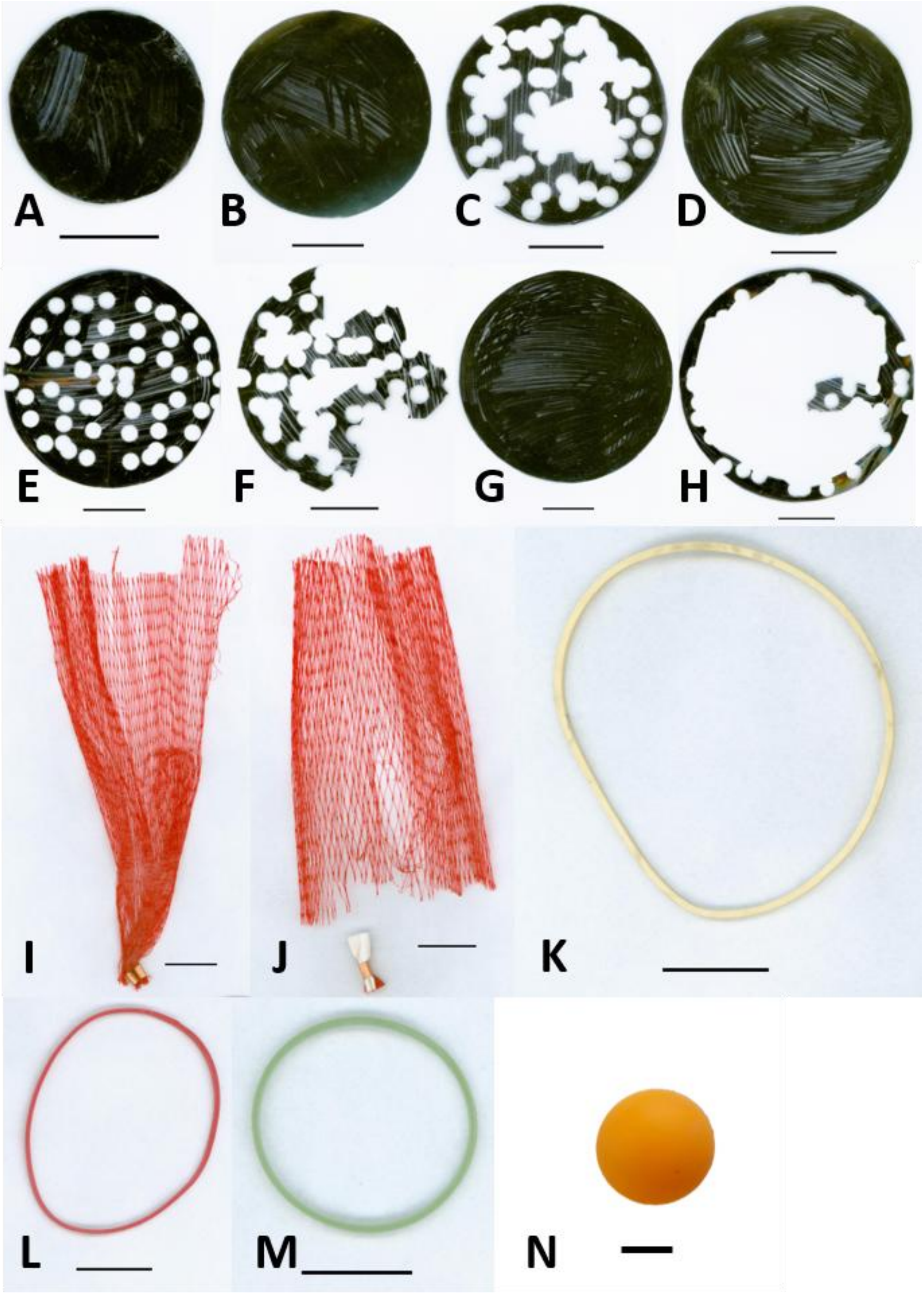

Fig. S8: Plastic particles. (A) Disc 40-1, (B) Disc 59-1, (C) Disc 59-2, (D) Disc 70-1, (E) Disc 70-2, (F) Disc 70-3, (G) Disc 84-1, (H) Disc 84-4, (I) Net-large, (J) Net-small, (K) Rubberband-large, (L) Rubberband-medium, (M) Rubberband-small, (N) Ball 1, 2, 3. Horizontal or vertical scale bars represent a distance of 2 cm.

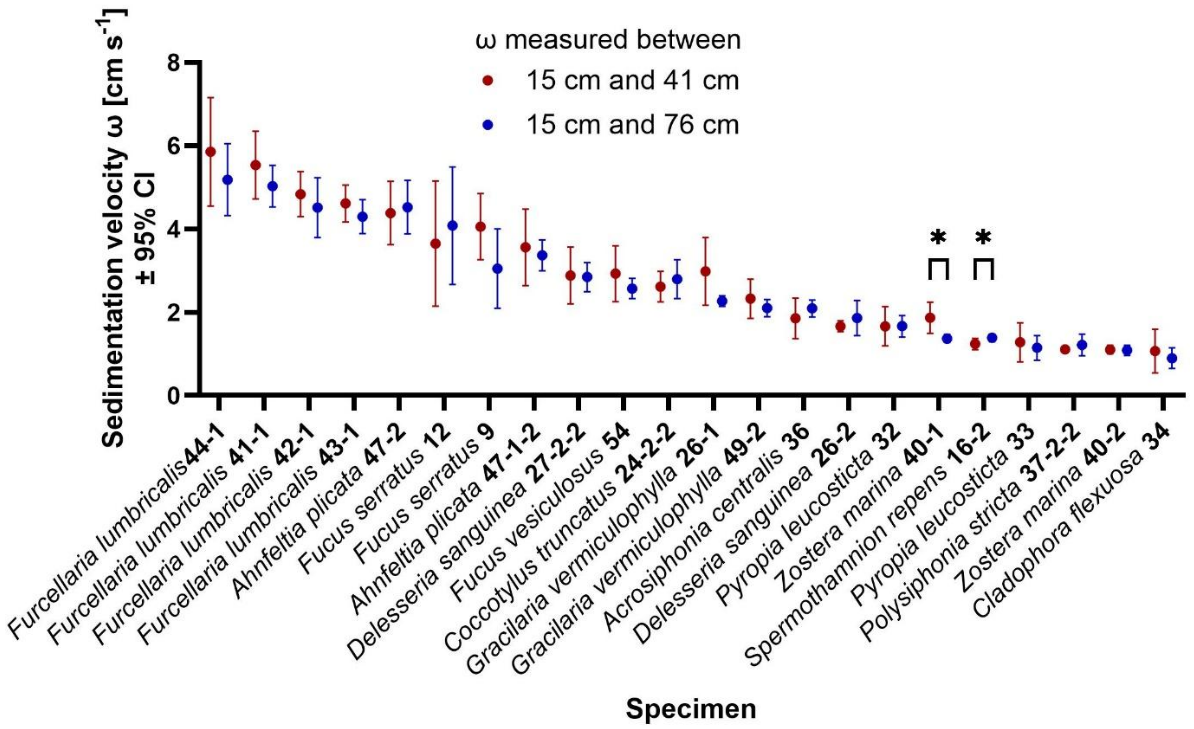

Fig S9: Comparison of sinking velocities of different macrophytes measured with two different methods. The specimens were released at the water surface and their velocity was measured after they had reached a depth of 15 cm. One approach measured ω in the water depth range between 15 cm and 41 cm, while the second measured it in the range between 15 cm and 76 cm. Asterisks indicate specimens for which significantly different results were obtained with the two approaches (Welch-corrected t-test, $n = 5$, $p < 0.05$).

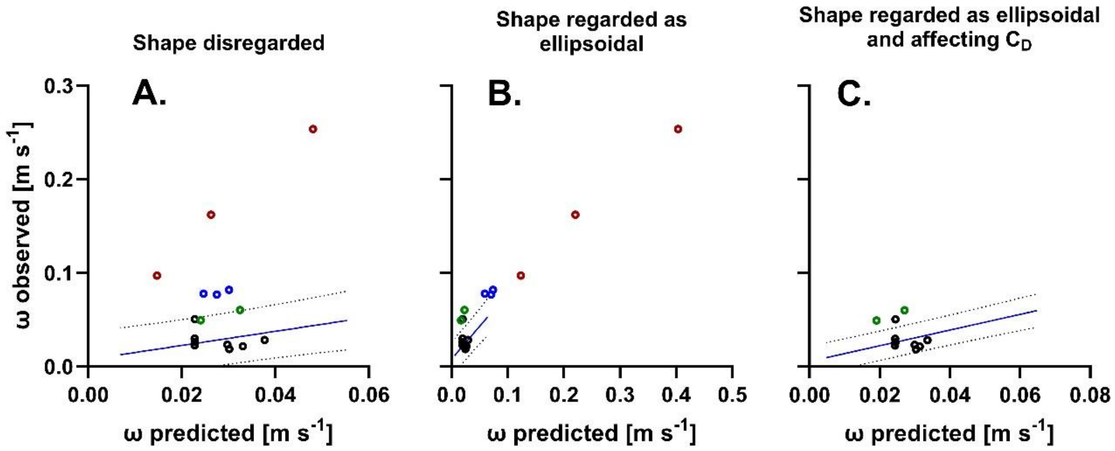

Fig. S10: Correlations of sedimentation velocities observed for plastic particles. Discs (black), nets (green), rubber bands (blue) and balls (red) with sedimentation velocities predicted for the same sample sets by (A) model A, (B) model B and (C) model C. In C data for balls and rubber bands are not shown, as predictions were extremely high (> 10^{99} m s^{-1}). Lines represent the linear functions that fitted best to data of the modeling macrophyte sample set (see also Figure 4), dotted lines represent 95 % prediction intervals.

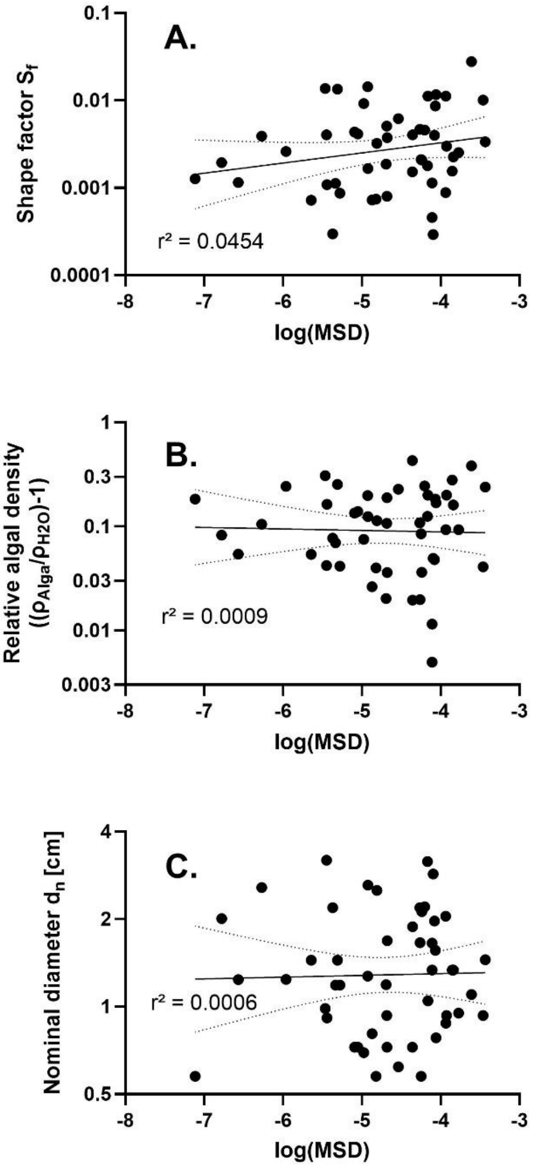

Fig S11: Double logarithmic correlations of morphological traits. (A) the shape factor, (B) the relative mass density and (C) the nominal diameter of 49 macrophyte specimens with the mean square deviation (MSD) of sinking velocities predicted by model C and observed with these specimens. Best fitting functions and their 95 % confidence intervals are shown (in all three cases $p > 0.05$).
